# A Short Review on the Model of Government Support Programs for Special Patients in Iran, United Kingdom, United States of America, Italy, and Sweden

**DOI:** 10.31661/gmj.v0i0.1403

**Published:** 2020-01-27

**Authors:** Esmaeil Hosseini, Mahmoud Mahmoudi Majd Abadi, Irvin Masoudi Asl, Behzad Karami Matin

**Affiliations:** ^1^Department of Health Service Administration, Science and Research Branch, Islamic Azad University, Tehran, Iran; ^2^Department of Health Service Management, School of Health Management and Information Sciences, Iran University of Medical Sciences, Tehran, Iran; ^3^Research Center for Environmental Determinants of Health, Kermanshah University of Medical Sciences, Kermanshah, Iran

**Keywords:** Supportive Programs, Special Patients, Financing, Organizational Structure, Control Mechanism

## Abstract

Over the past few decades, caring for special patients has taken center stage in healthcare systems. Moreover, what necessitates conducting a comparative study into the conditions of special patients and designing a suitable model are as follows: high admission rates of these patients in hospitals, continual recurrence of the illness, sky-high costs of treatment and medicine, lack of coordination between the services offered by hospitals and community needs, and severe pressure of special patients on their families. Therefore, the present study aimed to compare the models of government support programs for special patients in Iran, the United Kingdom, the USA, Italy and Sweden through a descriptive-documentary method. The findings revealed that the Ministry of Health and Welfare and the private sector were the major providers of health services to special patients in most of the countries under study. It was also demonstrated that the services offered to special patients are jointly mainly funded by governments, associations, and non-governmental organizations and partially by insurance premiums and so forth. The results also indicated that the bulk of healthcare provision was shouldered by non-governmental sectors and the contribution of charitable people. Finally, it can be concluded that both health-related policies and the health of special patients can be closely honed and monitored through the formation of committees on the health of special patients at the Supreme Council of Health, the establishment of an office for special patients at the Vice-chancellery for Health at the Ministry of Health, Treatment and Medical Education, provision of decentralized services, and financing through taxation and contribution of charitable people and international organizations.

## Introduction

Over the past few decades, caring for special patients has taken center stage in healthcare systems. Besides, what necessitates conducting a comparative study into the conditions of special patients and designing a suitable model are as follows: high admission rates of these patients in hospitals, continual recurrence of the illness, sky-high costs of treatment and medicine, lack of coordination between the services offered by hospitals and community needs, severe pressure of special patients on their families, and the patients’ families’ dissatisfaction with the quality of current services [1]. To overcome or minimize these issues, a social support program was introduced by Tenhoor and Turner (1998), in which special patients require more provisions than mere health services, and should they have access to comprehensive services, they can live their happy lives in societies [2]. Social support denotes the capability and quality of communication with others whereby some resources are supplied whenever required. In efforts made to understand the role of social support and its effects on health and well-being, two social support models have been investigated [3]. Social support plays efficacious roles in controlling diabetes through two major processes: the direct effects of social support through health-related behaviors, such as encouraging healthy behaviors, and its shield-like effects that help alleviate the effects of acute and chronic stress on health and increase the adaptation to the stress of diabetes [4]. Having studied the nursing texts, Morse *et al*. (2011) found out that the two concepts of care and support were similar in some aspects and different in some others [5]. Additionally, scholars believe that the concept, scope, and application of support to patients have not been clearly determined yet. However, Gardner and Wheeler (1981) claimed that support was a more detailed concept of care. They also reported that the concepts of support and its process had to be further investigated. Thus, the results of conducted studies are indicative of contradictory findings of these two concepts. Not to mention, despite the many supports by governmental and non-governmental organizations, there are still deep deficiencies in this area. According to the Ministry of Health of Iran, seven percent of people go below the poverty line because of the sky-high costs of treatment, but it can be easily predicted that the proportion of special patients in percentage is very escalating. Furthermore, the total amount of governmental and non-governmental supports is too insufficient to satisfy the financial needs of special patients who are often concerned about their unpredictable future and family breakdown [6]. Also, they often have financial problems, and some of the main effects of the disease on them are the various costs of treatment, travels to the health centers back and forth, drug provision, and so on. Besides, the occupational issues are other problems with which these patients are faced. In other words, as a result of their illness, these patients lose their occupational and educational opportunities, thereby losing their financial security and social status and facing adverse consequences due to the increasing costs of living [7]. The results of conducted studies in this field are indicative of various types of governmental and non-governmental supports provided to special patients. For example, in a study performed by Barry *et al*. (2006), the key variables that introduce the structure of a system to a large extent were first identified, and their relationships were then formulated in the form of cause and effect circles. By implementing the model, conducting sensitivity analysis and formulating the necessary policies, it will be clarified that how and to what extent the best possible conditions can be achieved so that the patients’ requirements are met and the governmental expenses are reduced [8]. The results of a study conducted by Lorenzo *et al*. (2010) revealed that there was a positive and significant relationship between social support and the ability to cope with breast cancer. Lorenzo *et al*. (2010) also reported that social interactions positively correlated with each of tangible, emotional, effective, informational, and emotional support. It was also demonstrated that the patients’ ability in dealing with their illnesses was positively and significantly related to the support from formal or informal sources and family sources separately [9]. In another study done by Faghani Aboukhalili *et al*. (2014), it was demonstrated that cancer survivors perceived high levels of social support, first from family members and then from certain individuals. It was also reported that the need for supportive care was decreased in all aspects as a result of growing social support [10]. In a study performed by Pourbaghi (2012) by the title of ‘the role of social support in improving the quality of life in hemodialysis patients,’ it was shown that hemodialysis patients received more emotional support than other types of support. Also, the respondents emphasized the priority of family as a source of social support. In Pourbaghi’s research, the theories proposed in previous studies on the role of social support in improving the quality of life in hemodialysis patients were confirmed and reinforced [11]. Therefore, given the adverse effects of the illness of special patients and the involvement of their families in their unpleasant situation, as well as the various effects of special illnesses on patients’ physical and psychosocial abilities to cope with the problem, the present study aimed to compare the models of government support programs for special patients in Iran, United Kingdom, USA, Italy, and Sweden.

## Special Diseases

The term ‘special diseases,’ first introduced by the Foundation for Special Diseases, refers to thalassemia, hemophilia, chronic renal failure, dialysis, and multiple sclerosis. Later on, thalassemia, hemophilia and dialysis disorders were approved by law under the title of ‘special diseases.’ There is no definition of ‘special diseases in common medicine, though [12]. Generally speaking, the characteristics of special diseases are as follows: severity of treatment, sky-high costs of treatment, and low prevalence. It should be noted that diseases with the said characteristics are known as rare diseases in different countries and Iran alike. However, as mentioned, thalassemia, hemophilia, and dialysis are exclusively known as special diseases in Iran, and treating these diseases is free of charge and covered by the Foundation for Special Diseases.

## Government Support

 Part of the main tasks of the government is to support the certain vulnerable social strata in the form of social security, which is one of the primary tasks that any country must realize. Therefore, since the government support programs on special patients are in the scope of the duties of social security, addressing the issues, and theoretical perspectives in this area is a necessity. Moreover, given that the establishment of social security services has strong theoretical foundations, it is necessary to explain this theory here briefly.

###  1. The United Kingdom Health System with an Emphasis on Special Patients

The United Kingdom health insurance system was founded in 1911, and half of its population at that time was covered by insurance, whose budget was provided by private insurers, trade unions, employers, and the State Insurance Committee. Later on, the National Health System (NHS) started its activities in 1948. At present, 82% of the NHS’s funding comes from taxes, 13% from employer-employee payments, and 4% from out-of-pocket payments. The responsibility for legislating and determining the overall health policies of the United Kingdom lies with the parliament and the Ministry of Health. Moreover, under the Social and Healthcare Act 2012, the responsibility for executing all the communication policies of the Ministry of Health was delegated to the National Public Service Agency. This organization, in charge of determining the budgets of the National Health Service, provides for the healthcare through its subsidiary bodies, including clinical service providers, health and welfare departments, local institutions, the National Institute for Health Excellence and Clinical Services, as well as its regional and local executive groups. Funding the health services mainly comes from the public finance and taxation, the National Insurance of Income Tax, and to some extent from the cost-sharing by patients and other sub-sources. Not to mention, all British residents are covered by a comprehensive healthcare system [13]. Furthermore, illegal tourists and refugees benefit exclusively from emergency medical services and care for certain infectious diseases for free. The health services covered by the National Health Service are as follows: preventive services (screening, immunization, and vaccination), general and specialized hospital and outpatient health services, mental health services, dentistry, rehabilitation, physiotherapy, long-term care, and nursing home visits.

####  1.1. Supervision of Support Programs

The Commission for the Quality of Healthcare is in charge of regulating all adult care and social care services in the United Kingdom. The National Health Service, local authorities, the private sector, and voluntary agencies, to name but a few, provide for such services. It should be noted that all healthcare providers such as institutions, individual partnerships, and physicians who are practicing individually and independently should be registered in the commission. Using quality standards at a national level, the commission monitors the performance of its members, and in the event of uncertainty in the quality of services (to patients), the providers are supervised, and poor and inadequate services are eliminated. Also, the National Institute for Health Promotion and Clinical Services shoulders the responsibility for regulating the quality of primary and secondary care and social support services. Besides, a special national strategy has been defined for certain conditions such as cancer, trauma, and stroke. Not to mention, major diseases and their key treatment procedures are categorized and registered based on some national criteria in agencies such as the National Cancer Registry Office and the National Linkage Registry Office. The health services covered by the National Health Service are as follows: preventive services (screening, immunization, and vaccination), general and specialized hospital and outpatient health services, mental health services, dentistry, rehabilitation, physiotherapy, long-term care, and nursing home visits [14].

####  1.2. Health Policy with an Emphasis on Special Diseases

 The key elements of the NHS model and social care for special diseases are as follows:

 - A systematic approach that best integrates the link between social care, health, patients and caregivers;

 - Identification of anyone with a special disease;

 - Categorization of people in such a way that the required care is received based on their requirements;

 - Focusing on the frequent consumers of secondary care services;

 - Using women in the community for providing case-based healthcare;

 - Developing methods for identifying people who may turn out to be using critical services;

 - Forming multidisciplinary teams in the field of primary healthcare under the supervision of an expert;

 - Developing the native practices of self-care through supporting them;

 - Developing expert-patient programs and other practices for promoting health management;

 - The application of effective tools and techniques that are currently available;

 - The long-term care model for the conditions of special patients is not quick and tacit last resort, but a multi-dimensional fix for a complex problem;

 - The four components of this model are more than anything else associated with a lasting change in service delivery and package offerings;

 - Organization of clinical teams;

 - Decision-making with the collaboration and participation of people suffering from special diseases;

- Encouraging providers to participate in promotion efforts [15].

###  2. The Sweden Health System with an Emphasis on Special Patients

 The private sector’s physicians and the comprehensive hospital services are the pivots on which the healthcare service in Sweden primarily turns. The main system of providing healthcare in Sweden is the Medicare System, which has been in place as a public health insurance system since 1984. Based on this system, all Swedish citizens have access to healthcare, medicine prescription and hospital care at approved prices. Not to mention, 70% of Sweden’s healthcare budget is incurred by the government. Sweden’s healthcare system consists of two public and private sectors, and the health system is administered in three categories:

 - National level or social welfare (central government);

 - State (six states) or regional level;

 - Local government.

 The Swedish healthcare system serves the public through three categories, from which the private sector plays a major role and the central government being at the top of the service delivery with precisely-defined tasks in each sector. Moreover, the private sector plays its vital role in this mechanism through private hospitals and private providers (clinics). Besides, decentralization of services has been done well, and the health services for mothers and children in need of mental care have been delegated to the states. Further, the responsibility for improving the environment is shouldered by municipalities and local administrations. The organizational structure of the Swedish health system is indicative of the existing priorities and issues and reflects the significance of the said categories. For instance, instead of the health of young Iranian population, special attention has been paid to the health of the elderly, so that the National Department of Health and the Elderly has been established in the central government of Sweden.

####  2.1. Financing the Health Services with an Emphasis on Special Diseases

The central governmental sector (public welfare) collects the majority of taxes, and the functional and financial responsibilities are divided among the state executives. It is worth mentioning that, with the cooperation of the private sector, the Swedish financing system aims to supply all citizens with access to all services with a right to choose, regardless of their financial ability and status (Table-1). The costs of inpatient and outpatient treatment are free in public hospitals, and medicine and dental care account for the major portion of the expenses. Medicare supplies all the essential health services and is financed by tax revenues, thereby covering all Swedish residents and incurring the costs of hospitals and medical services. Though the government regulates the tariffs, there is no limit for the income of doctors, coming either from the patients’ direct payments or the Health Insurance Commission (HIC). In the latter, around 85% of the fees are charged for the costs outside of hospitals whereas 75% of the fees go for the costs inside of hospitals. Moreover, the physicians get paid directly, and they are not allowed to receive extra money from the patients. Not to mention, about 75% of the family doctors send the medical bills directly to HIC. Should a doctor receive a tariff from the patient directly, the government shall reimburse the patient for the payment. It should be noted that the public hospitals, holding 70% of the hospital beds altogether, are run by the local government whereas only a few numbers of private hospitals enjoy emergency departments, which makes them entirely dependent on public hospitals. Also, Medicare’s public funding is spent on subsidizing medicines and giving grants to local governments and other centers. It is worth mentioning that 45% of the population use private insurance policies, thereby charging them somewhere in the neighborhood of $1,000-2,000 per household yearly. The insured receive their insurance coverage directly from the insurer and not the employer. Moreover, the premium is not risk-based; that is, when receiving a premium, the health status of the individual is not considered [16]. The health-related policies in the form of the Swedish federal government are determined by negotiations between the public welfare department and the states. Besides, the strategic direction of the Swedish health system is based on the use of more reliable and superior technologies to digitize all internal and external communication systems as well as the deployment of e-commerce. Therefore, the Swedish health system is implementing some technology and e-commerce programs and has taken some basic steps in this regard, including the digital processing of bills issued by doctors and pharmacies. This policy is in line with the implementation of a decision-making system for policymakers in the optimal allocation of resources because one of the basic infrastructures for implementing a coherent and integrated care system is the proper use of information technologies to collect data and key information in the health system. In addition, the quality of care offered to these patients is significantly influenced by the number and distribution of these patients in the country. In the Swedish health system, there is a plan known as ‘Pharmaceutical Benefits Scheme’ that subsidies the essential medicines, thereby charging special patients only $3 per prescription whereas other patients pay $22 per prescription. The program protects and screens security for the costs of each patient or family. Hence, after paying for the costs of 52 prescriptions, the retirees do not have to pay anything for other prescriptions until the end of the year. As for ordinary clients, after spending $686, they only pay $3 for each prescription for the rest of the year [17].

###  3. The Italian Health System with an Emphasis on Special Diseases 

Italy enjoys a public healthcare system that provides all its citizens and permanent residents with access to special care service. Although healthcare is administered by the ministry of health in provinces, the federal government has set some standards for healthcare across the country. Moreover, healthcare premiums are paid in three provinces (Ontario, Alberta, and British Columbia) whereas, in other provinces, the cost of treatment is paid by taxes and provincial governments. Italy has one of the most effective healthcare systems, and most of the healthcare services are available free of charge to all permanent residents and their families whose details have been registered under a national health insurance program called ‘Medicare,’ which pays for the costs of medical services provided by authorized doctors in hospitals and clinics. As an Italian citizen or a migrant with a sustainable status, you are entitled to all the benefits that Medicare offers. However, newly-arrived immigrants must wait for a certain period prior to requesting a sustainable status. Therefore, their treatment costs should be incurred by themselves by purchasing health travel insurance. With a unique national health insurance system integrated throughout the country, Italy has combined public and compulsory healthcare services with government funding on the one hand and somewhat private mechanisms for service delivery on the other hand. The Italian healthcare system is affected by the decentralized nature of its government, and according to the constitution, its ten states and three areas are responsible for the budgets, management and health services. However, the national government has major regulatory leverage and exercises its controlling role through its authority to prohibit federal funding for states when the required actions cannot be taken in accordance with a set of federal-level criteria. In 2000, Italy’s healthcare system ranked thirty, eighth and twelfth in terms of the overall health performance, satisfaction and responsiveness, and meeting the healthcare goals, respectively. Although the general satisfaction with the system has declined to 46%, Italy’s health system is still enjoying the highest popularity in the country for providing social programs [18]. Typically, the private health insurance in Italy is only applicable to services that are not covered by the government, which is often provided by employers in the form of supplementary health insurance as part of benefits in their own companies, thereby covering chronic diseases, dental/visual care, and non-medical services such as massage and physical therapies. In 1999, private health insurance policies were provided to 22.2 million Italians (73% of its population).

####  3.1. Health Policy with an Emphasis on Special Diseases 

The basis of the healthcare organization has been extensively determined in the Italian constitution, in which roles and responsibilities are shared among federal, state, and district governments, and the state and regional governments have the most responsibility in providing services to special patients, which are funded by the general government revenues. Three states of British Columbia, Alberta, and Ontario are also responsible for obtaining health insurance premiums. However, failure to pay premiums does not restrict access to essential medical services, and the healthcare services provided by the government create significant competitive advantages for the Italian market. Furthermore, the costs of health services are fairly distributed throughout the country by public funding, and the cost-effective tax system finances health insurance since no separate implementation process is required for revenue collection. Moreover, responsibilities for public health, such as healthy water supply sanitation, infectious disease control, and health literacy training, has been divided between three levels of government: federal, state / local, and municipal. However, as noted, the related services are generally provided at state/ regional and local levels [18].

####  3.2. Funding the Health Services for Special Patients in Italy

The funding and implementation obligations of the Italian health system are decentralized so that each state/region has its own insurance plans for residents who have resided for more than three months in that area. The role of the federal government is to monitor and empower the local/ state governments, and its power comes from its ability to prevent federal funding for health services. As a result, funding the states that cannot meet the five criteria stipulated in the Italian Health Act will be discontinued. However, services are supplied by private providers, either working individually as a physician or practicing clinically as a part of a medical group [18]. The main source of financial healthcare is tax administration by federal, state, and regional governments whereas the rest is provided by patients themselves in the form of out-of-pocket payments and private health insurance. Not to mention, the amount allocated to providers and health institutions is primarily provided by governments for a range of health goods and services subsidized by governments and subsequently supplied by consumers and patients individually for services and healthcare products in the private sector.

###  4. The U.S. Health System with an Emphasis on Special Diseases 

 With a population of 318 million, the USA of America has a gross domestic product of $52,610 and a total health expenditure of $8895. Additionally, the share of healthcare in the gross domestic product is 17.9%. Not to mention, the USA relies on private health insurance policies to fund healthcare and emphasizes the individual’s freedom and choice, but justice or equality is less prioritized. The elderly, the unemployed and the poor, who need more care, are not covered in the program, and the government does not have to finance these uninsured groups. Therefore, the Medicare coverage for health is funded by the federal government and Medicare is funded by the states to cover the poor. In this pluralistic and diverse system, about 43 million people still lack insurance coverage. The existence of various private health insurance plans weakens their bargaining power with providers and strengthens the ability of healthcare providers to gain exclusive benefits, which ultimately leads to a rise in health costs. To balance the market, buyer and seller power, many large firms are in favor of controlled competition, which requires sophisticated and advanced organizations to manage healthcare activities, where the administration costs can be very sky-high. Besides, it is not clear that this method, despite its success in reducing the excess supply of hospital beds in the USA in the 2000s, could control the growing health costs in the long-term. Not to mention, the USA does not have a coherent health system in terms of organizational structure because it consists of states that vary widely in terms of population, culture, and social customs. For this reason, they differ in terms of health needs and healthcare, and there are no comprehensive national systems for the health insurance system in the country, and the healthcare system has been formed by a complex combination of individual and public payers (federal, state, and local). Moreover, In the USA, the social security system has pledged itself to provide minimum healthcare for the vulnerable population. Therefore, health insurance coverage is optional for other people. The role of private insurance policies in the supply of health insurance is significant, and healthcare is provided with a variety of methods and qualities tailored to the choice of the insured. The financial management of the US Department of Health and Human Resources is responsible for managing federal health centers. Moreover, the state centers are mainly managed by municipalities, and the basic coverage of American health insurance includes a wide range of insurance programs that cover the costs of diseases, accidents, injuries and disabilities in both private and public sectors. More to the point, this wide range of health insurance includes medical costs, disability-income insurance, and accident insurance. In the USA, the responsibility for finance is divided between private insurance companies and the government, and they are both referred to as payers. Therefore, it is safe to say that a multi-payment system is used in the USA. Paying the costs of hospital services varies widely across the USA. For example, they can be paid through private insurers, federal Medicare state program, federal Medicare program, or even out-of-pocket payments. Third-party insurers pay hospitals for the offered services through various classified procedures, including retrospective and prospective payment systems, discounted rates, and so on. Insurance companies, healthcare organizations, Blue Cross, Blue Shield and other government agencies such as Medicare and Medicaid are third-party payers. The provision of health services to special patients is managed by the Center for Disease Control and Prevention. The center plays a role in providing various services within the framework of the care and prevention system for all diseases, including special, costly and non-curable diseases (chronic diseases).

###  5. Health Management of Special Diseases in Iran

In fact, the modern public health history in Iran goes back to the opening of *Dar-ul- Funun* in the Qajar era by *Mirza Taghi Khan Amir Kabir*. A French doctor by the name of Tholozan came to Tehran, the capital city of Iran, in 1864, who was both the physician of *Nasser-al-Din Shah* and a professor at *Dar-ul- Funun*. Later on, following frequent famine and epidemics of cholera, and with the suggestion of Dr. Tholozan, an organization was started by the title of ‘Hygiene House,’ locally known as ‘ *Hefzol Seha ’* at the time, which was the nation’s first public health organization. Moreover, the CEO of this organization was the Minister of Public Utilities, and its executive director was Dr. Tholozan. Not only did he found the hygiene house, but established a quarantine organization. In 1921, the title of the hygiene house was changed into the Supreme Council of Health, and later the Public Health Administration was established at the Ministry of Public Utilities, and the Supreme Council of Health was practically shut down, and finally, in 1941, the Ministry of Health was established. Not to mention, the public health department of the Ministry of Health in Iran began as an independent ministry in 1942. Later on, in 1946, the health plan became a big success, and it was in the same year that the statute of the World Health Organization was passed. However, its implementation was in 1948, and Iran is a member of this organization. It is worth mentioning that the provision of health services aimed at promoting, protecting and providing people with health is one of the important pillars of the progress of any society. Moreover, in the third, twenty-ninth and forty-third articles of the constitution of the Islamic Republic of Iran, the importance of providing healthcare is emphasized as the basic need of people because the health of the community is a means to human development [19]. In 1911, the first law of medicine was passed, and in February 1926, the health administration was established in accordance with the law of the centralization of health institutions of the country. Then, on October 29, 1941, the health administration was changed into the Ministry of Health. On July 23, 1950, the reduction of the administrative organization of the Ministry of Health was proposed by the Board of Appeal. Then, in 1948, according to the law, the transfer of healthcare to the people was approved. Thereafter, in June 1965, the focus and coordination of the healthcare affairs of the government employees were raised, which according to the Note 56 of the budget law of 1965, the institutions and therapeutic affairs of the Ministry of Health were assigned to Iran’s ‘ *Shir-o- Khorshid*.’ In 1966, the necessity of the detailed organization of ministries and institutions subject to the national employment law was adopted, which was approved in 1967. In July 1976, the Ministry of Health and Welfare was established, and on August 1, 1976, the regional health organizations of the provinces or the general governorate were formed. Besides, in 1979, the organization chart of the Ministry of Health and Welfare was established, too. Then, in October 1985, the Ministry of Health and Medical Education was formed, and the Ministry of Healthcare was dissolved, and the credits for universities and colleges of medicine and all higher education commitments in the medical group were separated and joined the Ministry of Health. Besides, in the sixth article of the law, it was allowed to create and develop new medical universities within the framework of higher education policies of the country by providing the necessary facilities and equipment. It also stipulates that the universities and faculties of medical sciences, like other universities, should be provided with an independent and separate budget annually. Therefore, the law of the organization and duties of the Ministry of Health was approved by the parliament on May 24, 1988. The law also emphasized the expansion of the integrated healthcare network, and the Ministry of Health was expanded with ten vice-chancelleries [20].

####  5.1. Funding Healthcare 

The main methods of financing in Iran include public funding, social insurance, and out-of-pocket payments made by the households. However, there are other methods, the most important of which is private insurance, which more often serves as a supplementary insurance policy for the current insured covered by the social insurances of health in Iran. Not to mention, private insurance policies now form a small portion of Iran’s health market. The main role of public funding in financing health services in Iran is focused on the coverage of health services. The country’s budget provides the country’s widespread healthcare system‒which is more comprehensive in rural areas‒and its services are also provided more by the public sector. However, the role of the state budget is not limited to healthcare services, and many second-level services, especially expensive treatments for special diseases, such as hemophilia, thalassemia, and alternative renal treatments, use public funds of the state. Additionally, the costs of the infrastructure of public hospitals are financed by the state budget, and state subsidies play a significant role in compensating for the costs of drug production [20]. Social insurance covers about 90% of the population for healthcare services such as outpatient, inpatient, and diagnostic services, and the level of coverage varies depending on the type of service and location, and in part, it depends on the insurance organization. For example, the health insurance mainly covers government employees and their family members the social insurance covers employees (other than government employees) and their family members, and the health insurance for armed forces which covers the personnel of armed forces and their family members. Not to mention, the premium is mainly paid by the employees’ monthly salary, of which the employer pays a larger share. There are numerous insurance funds covered by these organizations, and a large social insurance fund, known as ‘Rural Insurance Fund’ under the coverage of the Health Insurance Organization, has been established since 2005, as a result of which all residents in rural areas and small towns are covered. Besides, almost the entire premium of the members is paid by the state budget, and households and out-of-pocket payments incur a major portion of healthcare costs in Iran. In other words, more than half of the health costs in the country are paid directly by out-of-pocket payments [21].

####  5.2. Policies for Special Patient Health Services

Policies in the health sector are agreed at the national level and then notified to the provinces. The provincial planning committees, in accordance with the plans of the subsidiary counties, set up the provincial plans and submit it to the Planning Committee (healthcare), where the department’s overall programs are regulated whereas the detailed and executive planning is done in the provinces. Furthermore, the required credits regarding the program’s forecasts are provided to the counties through the province. Additionally, the executive information, as well as the data on health and well-being, are collected and communicated in the form of periodic reports to lower levels. It is worth mentioning that the Ministry of Health and Medical Education is in charge of policymaking in the health sector [18].

####  5.3. The Health System Structure with an Emphasis on Special Patients

 The integrated structure of health services in the country fits into three categories:

 1. Preventive or primary healthcare services are provided at a widespread level, mainly free of charge by the public health network available in cities and villages.

 2. Public and specialized outpatient care services provided by public and private centers.

 3. Hospital inpatient services: In this category, in addition to the Ministry of Health and Medical

**Figure 1:**
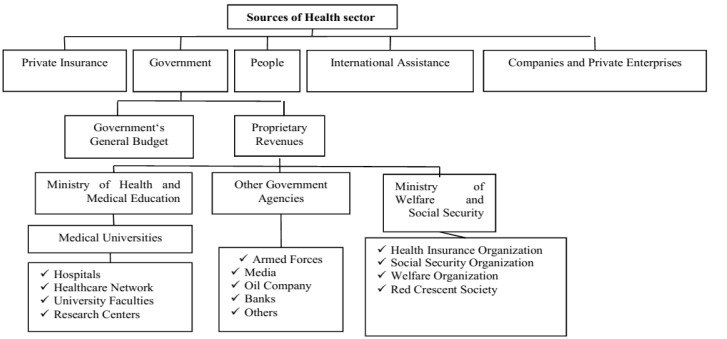
Healthcare finance providers and service providers in Iran

Education, social security and other government agencies, such as Army, Oil Companies, etc., other sectors such as private and cooperative sectors play significant roles in this respect [22]. In Figure-1, the overview of healthcare finance and service providers in Iran is presented.

###  6. Examination of the Models of Support Programs for Special Patients in Countries under Study

**Figure 2:**
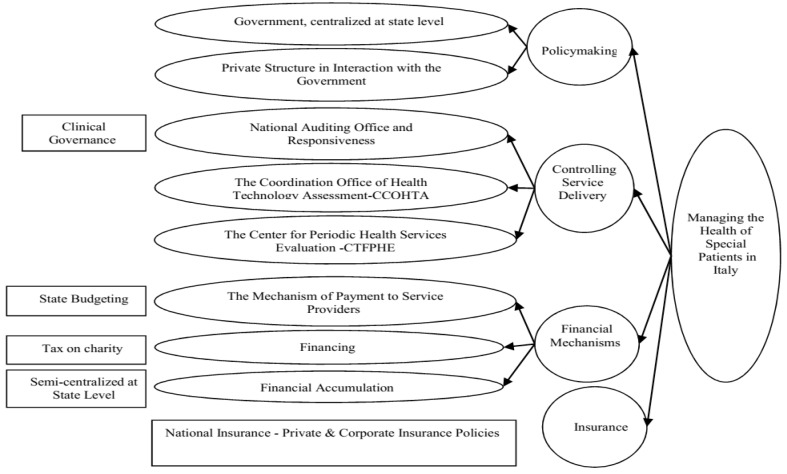
The healthcare model for special patients in Italy

**Figure 3:**
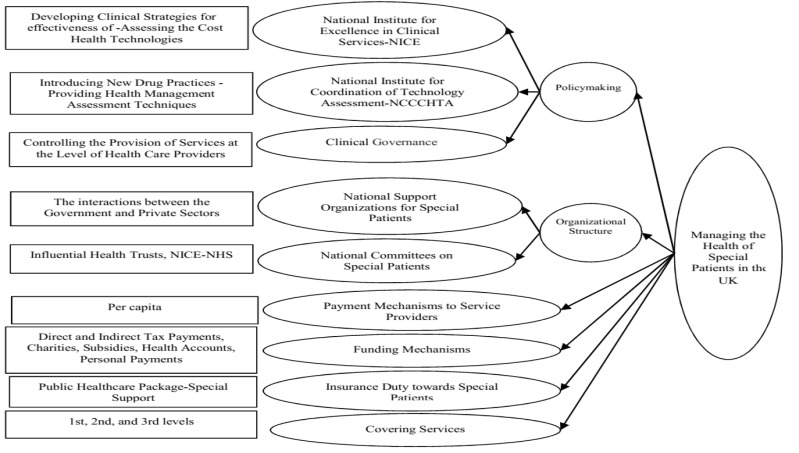
The healthcare model for special patients in the United Kingdom

**Figure 4:**
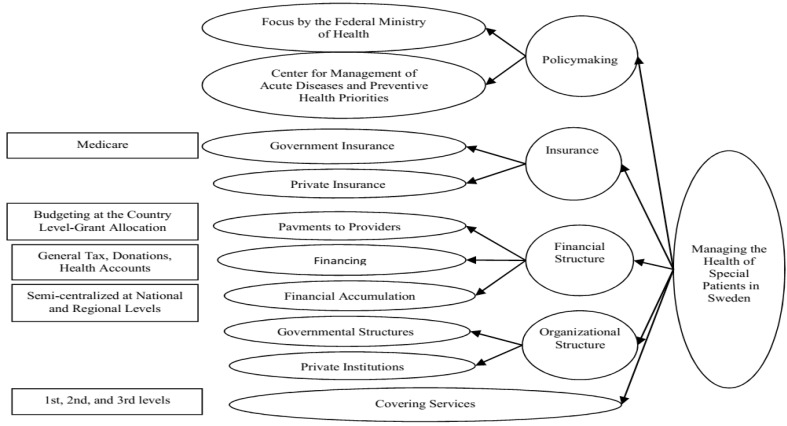
The healthcare model for special patients in Sweden

**Figure 5:**
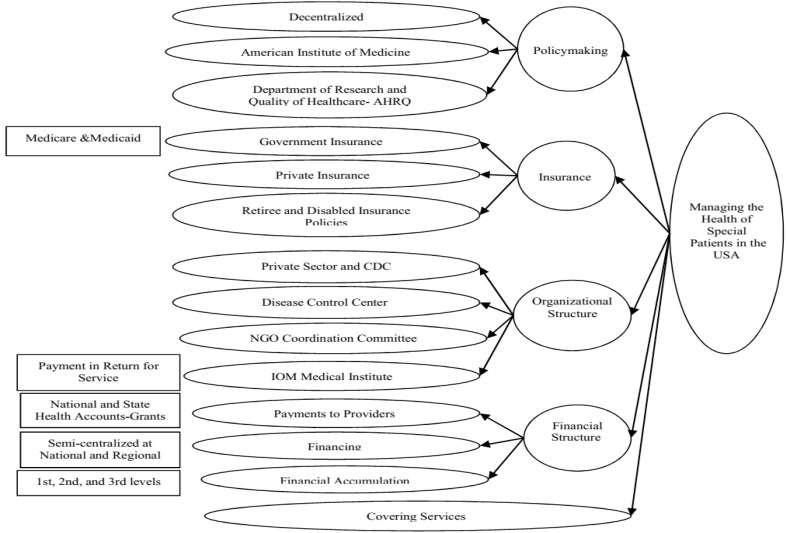
The healthcare model for special patients in the USA

The healthcare management in Italy enjoys four general scales: policymaking, control, funding mechanisms, and insurance services. Besides, there are nine sub-scales altogether, developed based on the country’s conditions, financial and cultural status, and its financial facilities and equipment (Figure-2) [3]. In contrast, the United Kingdom healthcare management system has six scales: policymaking, organizational structure, control, payment mechanisms to service providers, insurance duty towards special patients, funding mechanisms, and covering services. Moreover, there are 17 sub-scales altogether, developed according to the country’s conditions, financial and cultural status, and financial facilities and equipment (Figure-3) [23]. The Swedish healthcare management system has five scales: policymaking, organizational structure, financial structure, insurance services, and service coverage [24]. Also, there are 15 sub-scales altogether, developed in accordance with the country’s conditions, financial and cultural status, and financial facilities and equipment (Figure-4) [25]. Finally, the USA healthcare management system has five scales: policymaking, organizational structure, financial structure, insurance services, and service coverage. Further, there are 20 sub-scales altogether, developed in accordance with the country’s conditions, financial and cultural status, and financial facilities and equipment (Figure-5) [26].

## Conclusion

 The results of comparative studies demonstrated that the macro policies relating to the affairs of special patients in the countries under study were carried out by the Ministry of Health and Welfare or the highest health councils composing of representatives of ministries. Moreover, in Iran, the center for transplant management and special diseases is subject to many constraints by the Ministry of Health and Medical Education. The responsibility for legislating and determining the policies of health system management of chronic patients in the United Kingdom is assigned to the Parliament and the Ministry of Health. The organization, which is responsible for determining the budget of the NHS, provides health services to the covered individuals through its subsidiaries and regional and local executive groups. In Italy, public access to services is provided through a National Health Insurance Plan in which any citizen enjoys free healthcare, and national governments incur the required budgets and costs of national health insurance. In the USA, private health insurance policies are often sold to workers in the workplace to prevent reverse choices. However, the elderly, the unemployed and the poor, who need more care, are not covered. Therefore, the government should finance the insurance coverage of these groups. The federal government funds the Medicare coverage for the elderly and the states also fund Medicaid to cover the poor. In Sweden, the National Strategic Framework for Chronic Diseases has been in place since 2005 under the Sweden Health Ministers’ Advisory Council. In Sweden, there are public and private healthcare insurances with public access that are covered by a bilateral health service system. In Iran, the organizational position of chronic diseases at the level of the Ministry of Health and Medical Education is in the Department of Health and at the Center for the management of non-communicable diseases, and the national committee for the prevention and control of non-transmissible diseases was established in 2015. In the United Kingdom, the role of government in financing special patients is defined as providing services in the form of NHS and collecting taxes and insurance policies for employment assistance, and 11% of insurance policies are privately purchased for better access to healthcare services in private hospitals. In Sweden, the national and state sections have been merged by the government, national resources are provided for public hospitals, and the government manages the public health insurance program. In the USA, the people over the age of 65 through the Medicare insurance program and the disabled are covered by Medicare program whereas the people who do not have insurance coverage through their employers at the state level or have some exemptions are covered by Medicaid program (13.4% of adults are without insurance coverage). In Iran, the general budget is provided through the collection of public and private subsidies and allocations to the Ministry of Health through parliament. Therefore, the committee for the prevention and treatment of chronic patients is financed by the state. The results of comparative studies also revealed that in most of the countries under investigation, the Ministry of Health and Welfare and the private sectors were the major institutions for the provision of health services to special patients. Another point is that at present, there is no definite trustee for the provision of healthcare services to special patients in a transparent manner, and sometimes services are provided in parallel. Therefore, it is suggested that provision of healthcare services to special patients be done by public health-care units and the private sector with the cooperation and participation of the public. Finally, comparative studies showed that services provided to specific patients include services for all three levels of prevention: vaccination, teaching healthy lifestyle and disease care, publication of health leaflets, health counseling, health check services for early diagnosis and treatment of specific diseases, provision of auxiliary equipment for specific patients, laboratory services, home care and treatment, pharmacy and sanatorium services.

## Conflict of Interest

 There are no conflicts of interest.

**Table 1:** The Sources of Funding for the Swedish Healthcare System

** Financing Sources**	**Percentage of the Total**
Government	71.2
Private sector	16.3
Out-of-pocket payments	16.2
Private Insurance	7.1
Other institutions and funds	5.5

Source: (Research Writer)
